# 15-epi-lipoxin A_4_ reduces the mortality of prematurely born pups in a mouse model of infection-induced preterm birth

**DOI:** 10.1093/molehr/gau117

**Published:** 2015-01-07

**Authors:** S.F. Rinaldi, R.D. Catalano, J. Wade, A.G. Rossi, J.E. Norman

**Affiliations:** 1 MRC Centre for Reproductive Health and Tommy's Centre for Maternal and Fetal Health, University of Edinburgh, Queen's Medical Research Institute, 47 Little France Crescent, Edinburgh EH16 4TJ, UK; 2 MRC Centre for Inflammation Research, University of Edinburgh, Queen's Medical Research Institute, Edinburgh, UK

**Keywords:** anti-inflammatory, lipoxin, parturition, preterm birth, resolution

## Abstract

Preterm birth remains the leading cause of neonatal mortality and morbidity worldwide. There are currently few effective therapies and therefore an urgent need for novel treatments. Although there is much focus on trying to alter gestation of delivery, the primary aim of preterm birth prevention therapies should be to reduce prematurity related mortality and morbidity. Given the link between intrauterine infection and inflammation and preterm labour (PTL), we hypothesized that administration of lipoxins, key anti-inflammatory and pro-resolution mediators, could be a useful novel treatment for PTL. Using a mouse model of infection-induced PTL, we investigated whether 15-epi-lipoxin A_4_ could delay lipopolysaccharide (LPS)-induced PTL and reduce pup mortality. On D17 of gestation mice (*n* = 9–12) were pretreated with vehicle or 15-epi-lipoxin A_4_ prior to intrauterine administration of LPS or PBS. Although pretreatment with 15-epi-lipoxin A_4_ did not delay LPS-induced PTL, there was a significant reduction in the mortality amongst prematurely delivered pups (defined as delivery within 36 h of surgery) in mice treated with 15-epi-lipoxin A_4_ prior to LPS treatment, compared with those receiving LPS alone (*P* < 0.05). Quantitative real-time (QRT)-PCR analysis of utero-placental tissues harvested 6 h post-treatment demonstrated that 15-epi-lipoxin A_4_ treatment increased *Ptgs2* expression in the uterus, placenta and fetal membranes (*P* < 0.05) and decreased *15-Hpgd* expression (*P* < 0.05) in the placenta and uterus, suggesting that 15-epi-lipoxin A_4_ may regulate the local production and activity of prostaglandins. These data suggest that augmenting lipoxin levels could be a useful novel therapeutic option in the treatment of PTL, protecting the fetus from the adverse effects of infection-induced preterm birth.

## Introduction

Preterm labour (PTL), defined as labour before 37 weeks gestation, remains a major obstetric problem estimated to affect between 5 and 18% of pregnancies worldwide, with ∼15 million babies born prematurely each year (March of Dimes, 2012). Despite advances in the medical care of preterm infants, there are currently few effective treatment options and premature birth remains the leading cause of neonatal mortality. Indeed, preterm birth is estimated to account for up to 75% of neonatal deaths (Goldenberg *et al.*, 2008). Additionally, preterm birth is associated with an increased risk of a range of short-term morbidities and long-term disabilities, including cerebral palsy, bronchopulmonary dysplasia (BPD), retinopathy of prematurity and learning difficulties (Saigal and Doyle, 2008).

Spontaneous labour at term is now considered to be an inflammatory event that is associated with an immune cell infiltration into the cervix, myometrium and fetal membranes and increased production of pro-inflammatory mediators in the utero-placental tissues (Denison *et al.*, 1998; Thomson *et al.*, 1999; Sennstrom *et al.*, 2000; Young *et al.*, 2002; Osman *et al.*, 2003). Although the causes of PTL are often unclear, many cases are associated with the presence of occult or overt intrauterine infection (Goldenberg *et al.*, 2000) and the premature activation of these inflammatory pathways is likely responsible for PTL in this scenario. Animal models have confirmed a causal link between intrauterine infection and inflammation and PTL, given that injection of bacterial components, such as LPS or pro-inflammatory cytokines, such as tumour necrosis factor-α (TNF-α) or interleukin (IL)-1β reliably induce PTL (Romero *et al.*, 1991; Elovitz *et al.*, 2003; Sadowsky *et al.*, 2006). Our own *in vitro* studies have shown that LPS directly induces contractions of isolated human myometrial cells (Hutchinson *et al.*, 2013). Influx of immune cells likely also contributes to the process, although further work is required to define their precise roles (Timmons and Mahendroo, 2006; Murphy *et al.*, 2009; Thaxton *et al.*, 2009; Gonzalez *et al.*, 2011; Rinaldi *et al.*, 2014).

Given the link between inflammation and spontaneous labour onset, and the association between intrauterine infection and PTL, there has been a growing interest in examining whether anti-inflammatory agents could be effective novel therapeutic options for PTL (Rinaldi *et al.*, 2011). Animal studies have been invaluable in demonstrating the potential of a number of anti-inflammatory agents to delay preterm delivery and improve pup survival, including IL-10 (Terrone *et al.*, 2001; Rodts-Palenik *et al.*, 2004; Robertson *et al.*, 2006), TLR-4 signalling blockade (Adams Waldorf *et al.*, 2008; Li *et al.*, 2010), NFκB inhibitors (Nath *et al.*, 2010; Chang *et al.*, 2011) and 15-deoxy-Δ^12,14^-prostaglandin J_2_ (15d-PGJ_2_) (Pirianov *et al.*, 2009).

The understanding that the resolution of inflammation is an active process involving the production of mediators with specific anti-inflammatory and pro-resolution actions has provided new pathways to target in the search for novel treatments for inflammation-associated pathologies (Gilroy *et al.*, 2004; Serhan *et al.*, 2008). The arachidonic acid-derived lipid mediators, lipoxins, were the first family of mediators recognized to have dual-acting anti-inflammatory and pro-resolution actions (Serhan *et al.*, 1984; Ryan and Godson, 2010). During the resolution phase of an inflammatory response, in addition to the native lipoxins (lipoxin A_4_ and lipoxin B_4_), arachidonic acid can, in the presence of aspirin, also be converted to aspirin-triggered or 15-epi-lipoxins (15-epi-lipoxin A_4_ and 15-epi-lipoxin B_4_) (Chiang *et al.*, 2005). The anti-inflammatory and pro-resolution actions of lipoxins and 15-epi-lipoxins include: inhibiting neutrophil activation, adhesion and chemotaxis (Papayianni *et al.*, 1996; Filep *et al.*, 2005; Maderna and Godson, 2009); counteracting neutrophil anti-apoptotic signals (El Kebir *et al.*, 2007, 2009); triggering non-phlogistic phagocytosis of apoptotic neutrophils by macrophages (Godson *et al.*, 2000); stimulating monocyte adhesion and migration (Maddox and Serhan, 1996); and down-regulating pro-inflammatory cytokine production (Wu *et al.*, 2008; Kure *et al.*, 2009). The therapeutic potential of lipoxins has been widely demonstrated in animal models of a range of inflammation-associated pathologies, including asthma (Levy *et al.*, 2002) and lung injury (El Kebir *et al.*, 2009), arthritis (Zhang *et al.*, 2008; Conte *et al.*, 2010) and inflammatory bowel diseases (Gewirtz *et al.*, 2002). Within the reproductive tract, studies have identified a role for lipoxin signalling in endometriosis (Chen *et al.*, 2010; Xu *et al.*, 2012), embryo implantation (Xiong *et al.*, 2013) and spontaneous miscarriage (Xu *et al.*, 2013). To date, the role of lipoxins in regulating inflammation in parturition has been less well explored (Hutchinson *et al.*, 2011). However, using an *in vitro* model, we previously showed that expression of the lipoxin receptor, FPR2/ALX, is increased in myometrial tissue obtained from women during term labour (compared with tissue obtained from non-labouring women); and that lipoxin treatment down-regulated LPS-induced inflammatory gene expression in myometrial explant culture (Maldonado-Perez *et al.*, 2010).

Given evidence that lipoxins could be involved in regulating the inflammation associated with labour, and the therapeutic potential of lipoxin administration demonstrated in animal models of other inflammation-associated pathologies, we hypothesized that lipoxins could be effective therapeutic agents for the treatment of infection-induced PTL. In the study described here, using a mouse model of LPS-induced PTL, we evaluated the effect of pretreatment with 15-epi-lipoxin A_4_ on LPS-induced preterm delivery, pup mortality and the LPS-induced inflammatory response of the utero-placental tissues.

## Materials and Methods

### Mouse model of infection-induced PTL

All animal studies were conducted under a UK Home Office licence to JEN (60/4241) and were approved by the University's ethical board and the UK Home Office. Timed-pregnant CD-1 mice were obtained from Charles River Laboratories (Margate, UK) on D9-11 of gestation (the day vaginal plug was found was designated D1 of gestation). Mice were acclimatized for a minimum of 6 days prior to surgery. On D17 of gestation, a mini-laparotomy procedure was performed to expose the uterine horns, as previously described (Rinaldi *et al.*, 2014). The number of viable pups in each horn was recorded prior to injection. In LPS dose–response experiments, the horn with the greater number of fetuses was injected with either LPS (5–20 µg; from *Escherichia coli* 0111:B4; Sigma-Aldrich, Poole, UK) or sterile PBS (Gibco, Life Technologies Ltd, Paisley, UK) each in a 25 μl volume using a 33-gauge Hamilton syringe. Injections were performed directly into the uterine cavity between the first and second anterior fetuses. Care was taken not to enter any amniotic sacs. The wound was then closed and mice received a subcutaneous injection of Vetergesic analgesia (Alstoe Ltd, York, UK) at a dose of 0.03 mg/ml in 60 μl.

Mice were kept at 30°C while they recovered from surgery, before being transferred to individual cages for continuous monitoring using individual CCTV cameras and a digital video recorder. The time to delivery was recorded and defined as the number of hours from the time of intrauterine injection, to delivery of the first pup. Preterm delivery was defined as delivery of the first pup within 36 h of intrauterine injection. Term delivery in CD1 mice occurs on D19-21 of gestation, and we previously reported that mean (±SEM) time to delivery in a ‘no surgery’ control group of CD1 mice was 51.34 ± 1.13 h (*n* = 8), with all these mice delivering on D19 of gestation (Rinaldi *et al.*, 2014). Based on these data, delivery within 36 h of injection was chosen as preterm in our model. Within 12–24 h of delivery, the number of live/dead pups was recorded and the mortality rate per dam was calculated by dividing the number of dead pups by the number of viable pups counted *in utero* at the time of intrauterine injection.

In experiments to determine whether lipoxin administration could modulate LPS-induced preterm delivery and pup mortality, mice were pretreated with 15-epi-lipoxin A_4_ prior to intrauterine PBS or LPS administration. The 15-epi-lipoxin A_4_ analogue was chosen as several studies have reported that it is more stable, has a longer half-life *in vivo* and has more potent anti-inflammatory and pro-resolution effects, compared with lipoxin A_4_ (Serhan *et al.*, 1995; Serhan, 1997; Gewirtz *et al.*, 1998). Mice received an intra-peritoneal (i.p.) injection of vehicle (PBS + 1% ethanol) or 15-epi-lipoxin A_4_ (doses of 12.5 or 125 ng in a volume of 100 µl; Cayman Chemical, Ann Arbor, MI, USA), 1–2 h prior to intrauterine administration of PBS or 20 µg LPS. Therefore, there were five treatment groups: vehicle (i.p. injection of vehicle, followed by intrauterine PBS); 125 ng 15-epi-lipoxin A_4_ (i.p. injection of 125 ng 15-epi-lipoxin A_4_ followed by intrauterine PBS); LPS (i.p. injection of vehicle followed by intrauterine LPS); 12.5 ng 15-epi-lipoxin A_4_ + LPS (i.p. injection of 12.5 ng 15-epi-lipoxin A_4_ followed by intrauterine LPS) and 125 ng 15-epi-lipoxin A_4_ + LPS (i.p. injection of 125 ng 15-epi-lipoxin A_4_ followed by intrauterine LPS). The time to delivery and pup mortality rate was then recorded in each treatment group, as described earlier.

### Tissue collection

In a separate cohort of mice, to examine the effect of pretreatment with 15-epi-lipoxin A_4_ on the LPS-induced inflammatory response of the utero-placental tissues, tissues were collected 6 h post-surgery from mice treated with either vehicle or 15-epi-lipoxin A_4_ (0.25 and 2.5 µg) 1–2 h prior to intrauterine administration of PBS or 20 µg LPS. Higher doses of 15-epi-lipoxin A_4_ were used in these 6 h experiments to maximize the potential anti-inflammatory actions of 15-epi-lipoxin A_4_. All doses of 15-epi-lipoxin A_4_ used in this study were chosen based on published literature, which shows that lipoxins can be tolerated and have strong anti-inflammatory and pro-resolution effects over a wide range of doses *in vivo* (Levy *et al.*, 2002; El Kebir *et al.*, 2009; Kure *et al.*, 2009; Conte *et al.*, 2010; Borgeson *et al.*, 2011; Zhou *et al.*, 2011). Mice were culled by lethal exposure to CO_2_ and all pups were removed from the uterine horns and decapitated. Uterine tissue was sampled from three fixed sites within the uterus; fetal membranes were dissected free from the placenta, and these tissues were collected from three separate gestational sacs. Tissues were stored in RNA*later*^®^ (Sigma-Aldrich) at −80°C until processing.

### Quantitative real-time PCR

Total RNA was extracted from uterus, fetal membranes and placental tissue collected 6 h post-surgery using the RNeasy mini kit (Qiagen, Crawley, UK) as per the manufacturer's guidelines. Total RNA (300 ng) was reverse transcribed using the High Capacity cDNA Reverse Transcription kit (Applied biosystems, Life Technologies Ltd, Paisely, UK). Quantitative real-time PCR (qRT-PCR) was carried out to quantify the mRNA expression of specific genes of interest. Predesigned gene expression assays from Applied Biosystems were used to examine the expression of 15-hydroxy prostaglandin dehydrogenase (*15-Hpgd*) (Mm00515121_m1), *Il-10* (Mm00439614_m1), *Il-1β* (Mm00434228_m1), *Tnf-α* (Mm99999068_m1), *Cxcl1* (Mm04207460_m1), *Cxcl2* (Mm00436450_m1) and *Cxcl5* (Mm00436451_g1). Primer and probe sequences for *β-actin*, *Ptgs2* and *Il-6* were designed using Primer Express Software (version 3.0). Details of designed *β-actin*, *Ptgs2* and *Il-6* primer and probe sequences are given in Table I. Target gene expression was normalized for RNA loading using *β-actin* and the expression in each sample was calculated relative to a calibrator sample (untreated D18 uterus), which was included in all reactions, using the 2^−ΔΔCt^ method of analysis. All qRT-PCR analysis was performed on an Applied Biosystems 7900HT instrument.

**Table I:** Primer and probe sequences designed using Primer Express software.

Gene	Primer/Probe	Sequence
β-actin	Forward	5′-GCTTCTTTGCAGCTCCTTCGT-3′
	Reverse	5′-GCGCAGCGATATCGTCATC-3′
	Probe	5′-CACCCGCCACCAGTTCGCCAT-3′
Ptgs2	Forward	5′-GCTTCGGGAGCACAACAG-3′
	Reverse	5′-TGGTTTGGAATAGTTGCTC-3′
	Probe	5′-TGTGCGACATACTCAAGCA-3′
Il-6	Forward	5′-CCACGGCCTTCCCTACTTC-3′
	Reverse	5′-TGCACAACTCTTTTCTCATTCCA-3′
	Probe	5′-TCACAGAGGATACCACTCCCAACAGACCTG-3′

### Statistical analysis

Data are presented as mean ± SEM. Where data were not normally distributed they were transformed prior to analysis to achieve normal distribution. Time to delivery data was log-transformed before analysis; and the proportion of dead pups was arc-sin transformed prior to analysis. Data were analysed by one-way analysis of variance to compare treatment groups, followed by either Dunnett's or Newman–Keuls multiple comparison tests between treatment groups to identify significant differences. All statistical analyses were performed using GraphPad Prism 5.0 software (Graph Pad, San Diego, CA, USA). *P* < 0.05 was considered to indicate statistical significance.

## Results

### Intrauterine LPS administration dose-dependently increases pup mortality

**Figure 1:**
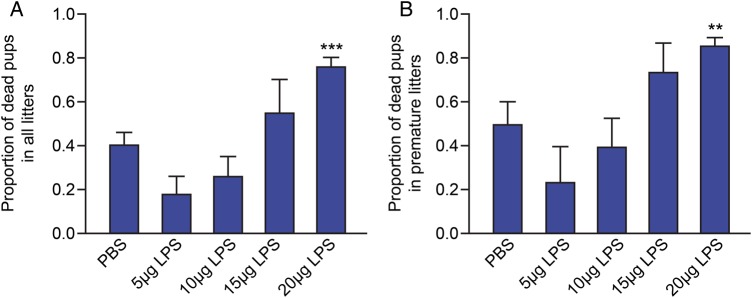
Effect of intrauterine LPS administration on pup mortality. The proportion of dead pups were determined in mice receiving intrauterine injection of either phosphate-buffered saline (PBS; *n* = 35), 5 μg LPS (*n* = 6), 10 μg LPS (*n* = 11), 15 μg LPS (*n* = 8) and 20 μg LPS (*n* = 42). (**A**) Proportion of dead pups in all litters. (**B**) Proportion of dead pups in premature litters (delivered within 36 h of surgery); [PBS (*n* = 14), 5 μg LPS (*n* = 3), 10 μg LPS (*n* = 6), 15 μg LPS (*n* = 6) and 20 μg LPS (*n* = 35)]. Data presented as mean ± SEM (error bars); **P < 0.01, ***P < 0.001, compared with PBS.

As we have previously reported, intrauterine LPS administration dose-dependently induces PTL in a mouse model (Rinaldi *et al.*, 2014). To assess the effects of intrauterine LPS treatment on pup mortality mice were treated with increasing doses of LPS and the pup mortality rate was calculated following delivery. Pup mortality was increased in response to administration of increasing doses of intrauterine LPS, with a significantly higher proportion of dead pups born to mice treated with 20 µg LPS, compared with the PBS control group (mean ± SEM proportion of dead pups 0.75 ± 0.05 versus 0.40 ± 0.06, respectively, *P* < 0.001; Fig. 1A). To further investigate whether this observed increase in pup mortality in the 20 µg LPS group was simply due to a higher proportion of preterm deliveries in this group, rather than a direct effect of the LPS treatment, pup mortality was also assessed only in mice delivering preterm (defined as delivery within 36 h of surgery). Even amongst mice delivering preterm, fetal mortality was still significantly greater in mice treated with 20 µg LPS group compared with PBS (mean ± SEM proportion of dead pups 0.85 ± 0.04 versus 0.49 ± 0.11, respectively, *P* < 0.01; Fig. 1B). Subsequent experiments were performed with 20 µg LPS as this dose has been shown to induce preterm delivery reliably in our model with the least variation (Rinaldi *et al.*, 2014).

### Pretreatment with 15-epi-lipoxin A_4_ reduces pup mortality without delaying LPS-induced preterm delivery

**Figure 2:**
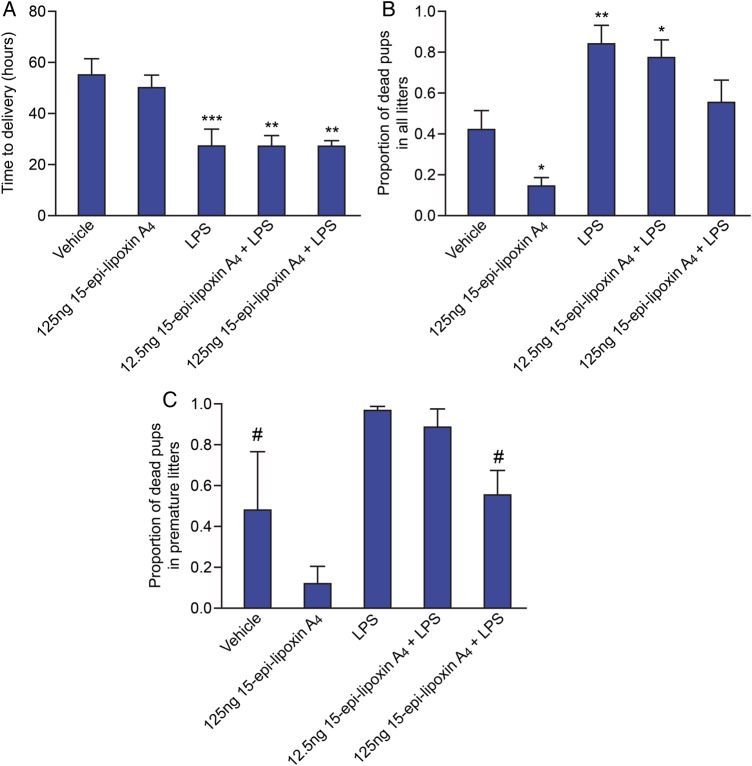
Effect of pretreatment with 15-epi-lipoxin A_4_ on time to delivery and pup mortality. Time to delivery and the proportion of dead pups was determined in mice pretreated with vehicle (*n* = 12) or 125 ng 15-epi-lipoxin A_4_ (*n* = 9), prior to intrauterine PBS; and in mice pretreated with vehicle (*n* = 12), 12.5 ng 15-epi-lipoxin A_4_ (*n* = 11) or 125 ng 15-epi-lipoxin A_4_ (*n* = 11), prior to intrauterine LPS (20 µg) administration. (**A**) Time to delivery. (**B**) Proportion of dead pups in all litters. (**C**) Proportion of dead pups in premature litters (delivered within 36 h of surgery); [Vehicle (*n* = 3), 125 ng 15-epi-lipoxin A_4_ (*n* = 2), LPS (*n* = 10), 12.5 ng 15-epi-lipoxin A_4_ (*n* = 7) or 125 ng 15-epi-lipoxin A_4_ (*n* = 10)]. The 15-epi-lipoxin A_4_ group was excluded from statistical analysis of the proportion of prematurely delivered dead pups due to *n* < 3. Data presented as mean ± SEM (error bars); *P < 0.05, **P < 0.01, ***P < 0.001, compared with vehicle; ^#^*P* < 0.05 compared with LPS.

To investigate the therapeutic potential of lipoxin to delay preterm delivery and reduce prematurity induced fetal mortality, mice were pretreated with 15-epi-lipoxin A_4_ 1–2 h prior to intrauterine LPS (20 µg) or PBS administration. Control mice were pretreated with vehicle prior to intrauterine LPS or PBS administration. Pretreatment with 125 ng 15-epi-lipoxin A_4_ prior to intrauterine PBS had no effect on time to delivery compared with the vehicle control group (Fig. 2A). As expected mice receiving intrauterine LPS delivered significantly earlier than the vehicle control group (LPS mean time to delivery: 27.54 h ± SEM 6.33; versus vehicle mean time to delivery: 55.40 h ± SEM 6.40; *P* < 0.001; Fig. 2A). Pretreatment with either 12.5 or 125 ng 15-epi-lipoxin A_4_ prior to intrauterine LPS administration did not delay LPS-induced PTL. Mice in these groups still delivered significantly earlier than the vehicle control group (mean ± SEM time to delivery 12.5 ng 15-epi-lipoxin A_4_ + LPS: 27.02 ± 4.57 h; mean time delivery in 125 ng 15-epi-lipoxin A_4_ + LPS: 26.82 ± 2.61; *P* < 0.01 versus vehicle).

Again as expected, mice treated with LPS alone had significantly increased pup mortality compared with the vehicle group (mean ± SEM proportion of dead pups: 0.84 ± 0.09; *P* < 0.01; Fig. 2B). Interestingly, pretreatment with 125 ng 15-epi-lipoxin A_4_ prior to intrauterine PBS significantly reduced pup mortality, compared with the vehicle control group (mean ± SEM proportion of dead pups 0.13 ± 0.05 versus 0.42 ± 0.1, respectively, *P* < 0.05; Fig. 2B). Within the subgroup of mice delivering preterm (within 36 h of surgery), pretreatment with 125 ng 15-epi-lipoxin A_4_ prior to intrauterine LPS significantly reduced pup mortality, compared with mice receiving LPS alone (mean proportion ± SEM of dead pups 0.55 ± 0.12 versus 0.97 ± 0.02, respectively; *P* < 0.05; Fig. 2C).

### Pretreatment with 15-epi-lipoxin A_4_ alters the expression of Ptgs2 and 15-Hpgd in the utero-placental tissues, but does not attenuate LPS-induced expression of classical pro-inflammatory markers

To examine whether pretreatment with 15-epi-lipoxin A_4_ affected the LPS-induced inflammatory response of the utero-placental tissues, qRT-PCR analysis was performed on uterus, placenta and fetal membranes collected 6 h post-surgery. The mRNA expression of several key inflammatory genes associated with parturition was quantified. The genes measured were: two of the key enzymes responsible for regulating prostaglandin synthesis and breakdown, respectively, *Ptgs2* and *15-Hpgd*; the pro-inflammatory cytokines *Il-1β*, *Tnf-α* and *Il-6*; and the chemokines *Cxcl1*, *Cxcl2* and *Cxcl5*. To investigate the anti-inflammatory actions of 15-epi-lipoxin A_4_, we administered higher doses (0.25 and 2.5 µg) of 15-epi-lipoxin A_4_ 1–2 h prior to LPS or vehicle to try to maximize the anti-inflammatory effects in these 6 h experiments. As stated earlier, all doses of 15-epi-lipoxin A_4_ used were within the range of effective doses used *in vivo* in previously published studies.

**Figure 3:**
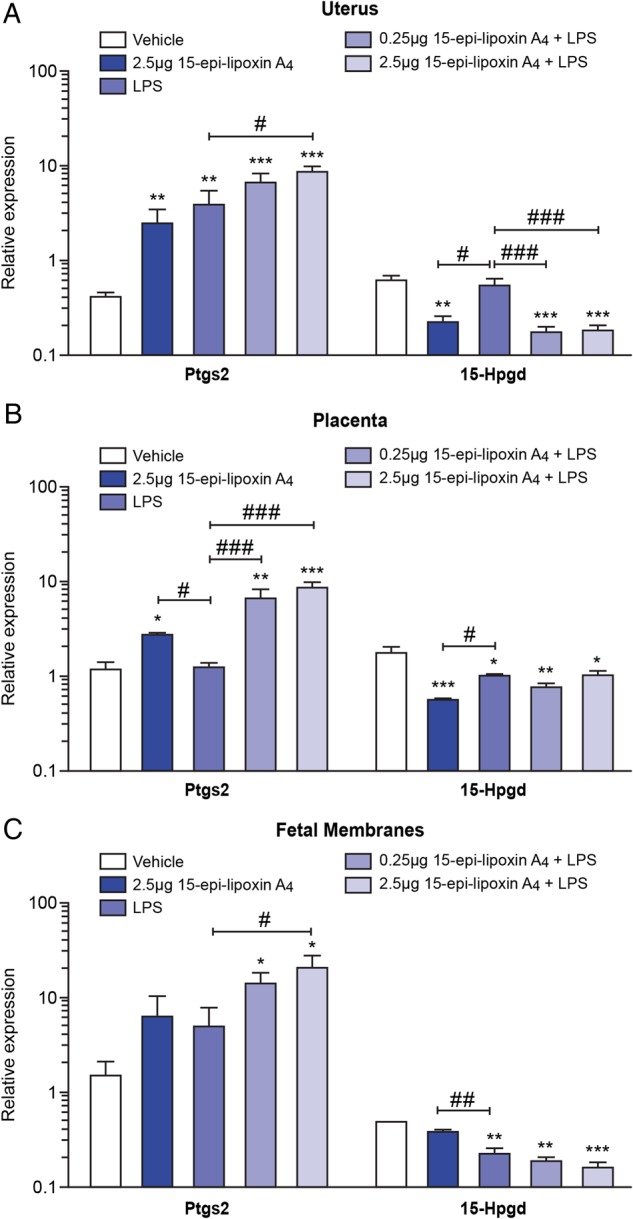
Effect of pretreatment with 15-epi-lipoxin A_4_ on mRNA expression of *Ptgs2* and *15-Hpgd* in the utero-placental tissues. Uterus, placenta and fetal membranes were collected 6 h post-surgery from mice pretreated with vehicle (*n* = 3) or 2.5 μg 15-epi-lipoxin A_4_ (*n* = 4), prior to intrauterine PBS; and in mice pretreated with vehicle (*n* = 5), 0.25 μg 15-epi-lipoxin A_4_ (*n* = 5) or 2.5 μg 15-epi-lipoxin A_4_ (*n* = 5), prior to intrauterine LPS administration. The mRNA expression of *Ptgs2* and *15-Hpgd* was quantified by quantitative real-time PCR. (**A**) Uterine expression. (**B**) Placental expression. (**C**) Expression in the fetal membranes. Data presented as mean fold change ± SEM (error bars); *P < 0.05, **P < 0.01, ***P < 0.001, compared with vehicle; ^#^*P* < 0.05, ^##^*P* < 0.01, ^###^*P* < 0.001, compared with LPS.

In the uterus, *Ptgs2* expression was significantly elevated in response to 2.5 µg 15-epi-lipoxin A_4_ alone (*P* < 0.01), LPS alone (*P* < 0.01), and 0.25 µg and 2.5 µg 15-epi-lipoxin A_4_ + LPS (*P* < 0.001; Fig. 3A), compared with the vehicle control group. Co-treatment with 2.5 µg 15-epi-lipoxin A_4_ and LPS also significantly increased uterine *Ptgs2* expression compared with treatment with LPS alone (*P* < 0.05; Fig. 3A). Conversely, uterine *15-Hpgd* expression was significantly reduced in mice treated with 2.5 µg 15-epi-lipoxin A_4_ prior to intrauterine PBS, compared with vehicle (*P* < 0.01) and LPS alone (*P* < 0.05). LPS alone did not significantly alter *15-Hpgd* expression; however, mice treated with 0.25 µg 15-epi-lipoxin A_4_ + LPS and 2.5 µg 15-epi-lipoxin A_4_ + LPS had significantly reduced uterine *15-Hpgd* expression, compared with the vehicle group (*P* < 0.001). Additionally pretreatment with 0.25 µg 15-epi-lipoxin A_4_ and 2.5 µg 15-epi-lipoxin A_4_ prior to intrauterine LPS, significantly reduced uterine expression of *15-Hpgd*, compared with LPS alone (*P* < 0.001; Fig. 3A).

Placental *Ptgs2* expression was significantly elevated in mice treated with 2.5 µg 15-epi-lipoxin A_4_ prior to intrauterine PBS, compared with vehicle (*P* < 0.05; Fig. 3B) and LPS alone (*P* < 0.05). *Ptgs2* expression in the placenta was unaffected by LPS alone, but pretreatment with 15-epi-lipoxin A_4_ at both 0.25 and 2.5 µg prior to intrauterine LPS administration significantly increased *Ptgs2* expression compared with both the vehicle control group (*P* < 0.01 and *P* < 0.001, respectively; Fig. 3B) and compared with LPS treatment alone (*P* < 0.001; Fig. 3B). Placental *15-Hpgd* expression was significantly down-regulated in response to 2.5 µg 15-epi-lipoxin A_4_ alone (*P* < 0.001), LPS alone (*P* < 0.05), 0.25 µg 15-epi-lipoxin A_4_ + LPS (*P* < 0.01) and 2.5 µg 15-epi-lipoxin A_4_ + LPS (*P* < 0.05; Fig. 3B), compared with the vehicle control group.

In the fetal membranes, intrauterine LPS treatment alone did not significantly alter *Ptgs2* expression; however, mice treated with 0.25 µg 15-epi-lipoxin A_4_ + LPS had significantly elevated *Ptgs2* expression compared with the vehicle control group (*P* < 0.05; Fig. 3C); and mice treated with 2.5 µg 15-epi-lipoxin A_4_ + LPS had significantly elevated *Cox-2* expression, compared with both the vehicle control group and LPS alone (*P* < 0.05; Fig. 3C). Expression of *15-Hpgd* in the fetal membranes was significantly reduced in response to LPS treatment alone (*P* < 0.01), 0.25 µg 15-epi-lipoxin A_4_ + LPS (*P* < 0.01) and 2.5 µg 15-epi-lipoxin A_4_ + LPS (*P* < 0.001; Fig. 3B).

**Figure 4:**
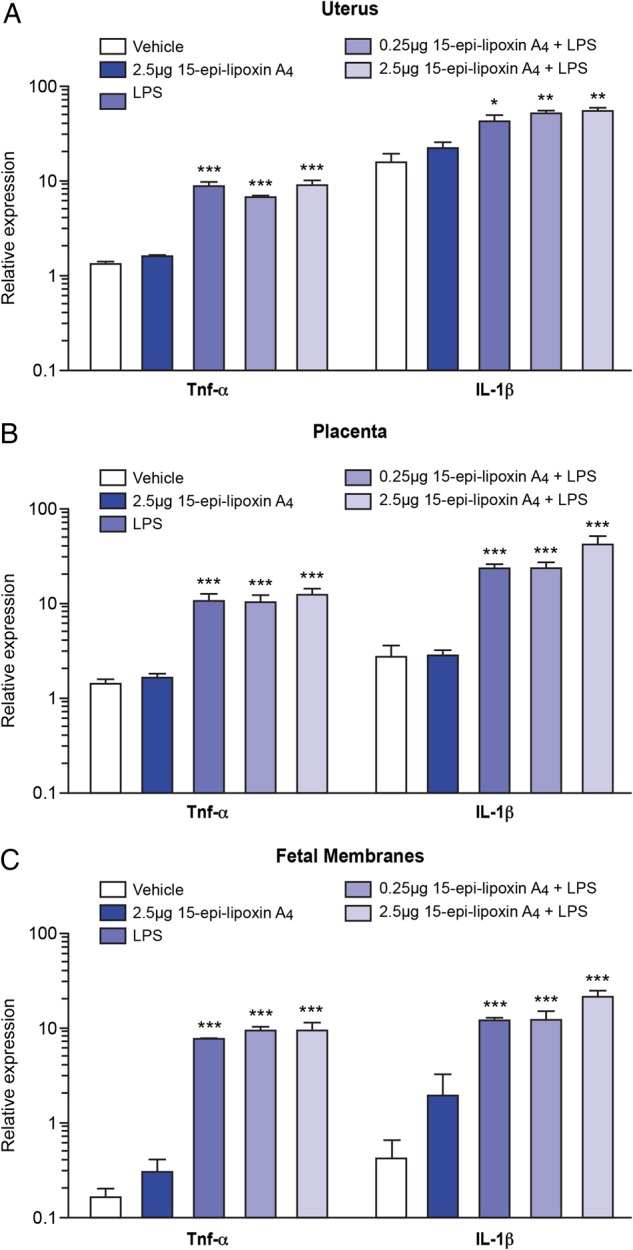
Effect of pretreatment with 15-epi-lipoxin A_4_ on mRNA expression of *Tnf-α* and *Il-1β* in the utero-placental tissues. Uterus, placenta and fetal membranes were collected 6 h post-surgery from mice pretreated with vehicle (*n* = 3) or 2.5 μg 15-epi-lipoxin A_4_ (*n* = 4), prior to intrauterine PBS; and in mice pretreated with vehicle (*n* = 5), 0.25 μg 15-epi-lipoxin A_4_ (*n* = 5) or 2.5 μg 15-epi-lipoxin A_4_ (*n* = 5), prior to intrauterine LPS administration. The mRNA expression of *Tnf-α* and *Il-1β* was quantified by quantitative real-time PCR. (**A**) Uterine expression. (**B**) Placental expression. (**C**) Expression in the fetal membranes. Data presented as mean fold change ± SEM (error bars); *P < 0.05, **P < 0.01, ***P < 0.001, compared with vehicle.

In contrast to the effects on *Ptgs2* and *15-Hpgd*, pretreatment with 15-epi-lipoxin A_4_ at either 0.25 or 2.5 µg prior to intrauterine LPS did not attenuate or amplify the LPS-induced expression of the classical inflammatory markers *Tnf-α* and *Il-1β* in the uterus (Fig. 4A), placenta (Fig. 4B) and fetal membranes (Fig. 4C). Similarly, pretreatment with 15-epi-lipoxin A_4_ did not alter the LPS-induced expression of the other inflammatory mediators examined, *Il-6*, *Cxcl1*, *Cxcl2* and *Cxcl5* (data not shown).

### Pretreatment with 15-epi-lipoxin A_4_ does not further up-regulate the LPS-induced expression of Il-10 in the utero-placental tissues

**Figure 5:**
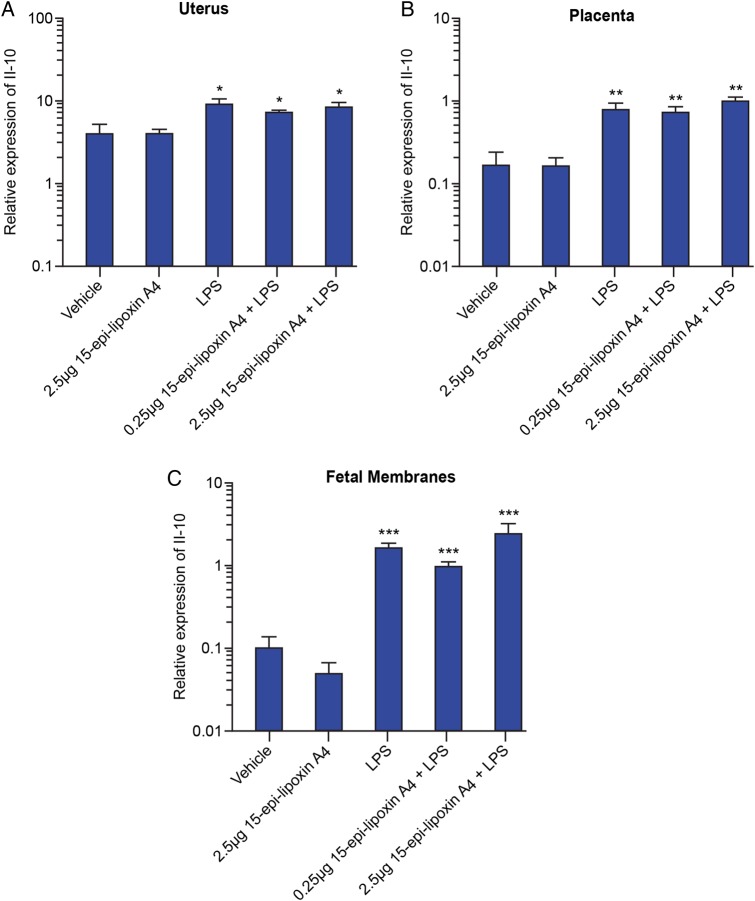
Effect of pretreatment with 15-epi-lipoxin A_4_ on mRNA expression of *Il-10* in the utero-placental tissues. Uterus, placenta and fetal membranes were collected 6 h post-surgery from mice pretreated with vehicle (*n* = 3) or 2.5 μg 15-epi-lipoxin A_4_ (*n* = 4), prior to intrauterine PBS; and in mice pretreated with vehicle (*n* = 5), 0.25 μg 15-epi-lipoxin A_4_ (*n* = 5) or 2.5 μg 15-epi-lipoxin A_4_ (*n* = 5), prior to intrauterine LPS administration. The mRNA expression of *Il-10* was quantified by quantitative real-time PCR. (**A**) Uterine expression. (**B**) Placental expression. (**C**) Expression in the fetal membranes. Data presented as mean fold change ± SEM (error bars); *P < 0.05, **P < 0.01, ***P < 0.001, compared with vehicle.

Previous studies have reported that one mechanism by which lipoxins exert anti-inflammatory actions is by up-regulating the expression of the anti-inflammatory cytokine, IL-10 (Baker *et al.*, 2009; Borgeson *et al.*, 2011). Therefore, we investigated the mRNA expression of *Il-10* in the utero-placental tissues 6 h post-surgery using qRT-PCR. Treatment with LPS alone resulted in significantly elevated expression of *Il-10* in the uterus (*P* < 0.05; Fig. 5A), placenta (*P* < 0.01; Fig. 5B) and fetal membranes (*P* < 0.001; Fig. 5C). However, pretreatment with 15-epi-lipoxin A_4_, at either 0.25 or 2.5 µg, prior to intrauterine LPS treatment did not result in a further increase in *Il-10* expression, compared with LPS alone.

## Discussion

We have previously shown the anti-inflammatory effects of the dual-acting anti-inflammatory and pro-resolution lipid mediators, lipoxins, in human gestational tissues *in vitro* (Maldonado-Perez *et al.*, 2010). Here, we tested the efficacy of 15-epi-lipoxin A_4_ as a novel therapeutic agent in an *in vivo* mouse model of infection-induced PTL. Contrary to our original hypothesis, we did not observe a reduction in preterm delivery or reduced pro-inflammatory signalling in mice treated with 15-epi-lipoxin A_4_. We did, however, show that 15-epi-lipoxin A_4_ treatment reduced the mortality of prematurely delivered pups and altered basal and LPS-induced *Ptgs2* and *15-Hpgd* expression in the utero-placental tissues.

We believe that the finding that 15-epi-lipoxin A_4_ treatment resulted in a greater proportion of prematurely delivered pups being born alive is a novel and important discovery. Current treatment options for preterm birth are largely limited to tocolytic therapies that aim to block myometrial contractions and prolong gestation. However, there is little convincing evidence that these treatments actually result in improved neonatal outcome in the long-term. Given that preterm birth remains the single biggest cause of neonatal mortality and morbidity worldwide, there is an urgent requirement for novel therapeutic options which are capable of achieving the ultimate goal of preterm prevention therapies—reduced perinatal mortality and morbidity. Interestingly a recent paper has highlighted the potential of lipoxin treatment to treat the preterm-related lung disease, BPD (Martin *et al.*, 2014). Using a mouse model of hyperoxia-induced lung injury Martin *et al.* (2014) reported that lipoxin A_4_ treatment given (post-natally) to neonatal pups reduced the morphologic and cellular characteristics of lung injury and improved pup growth; therefore, supporting the hypothesis that pre- and post-natal lipoxins could be useful novel therapeutic agents to improve neonatal outcome.

The pup mortality observed in our model is likely to be a result of the immaturity of the prematurely delivered pups, which if delivered on Day 17 or 18 of gestation are unlikely to be developmentally competent to survive, and also the LPS treatment given to the mice. Owing to the invasive nature of the model, which we have previously shown results in a local inflammatory response, even in mice treated with PBS (Rinaldi *et al.*, 2014), some of the control mice do deliver prematurely, and therefore do experience some pup mortality. We are currently exploring other, less invasive methods, to reduce this preterm delivery in our control group. Importantly, however, we did observe a significant reduction in pup mortality in mice treated with intrauterine PBS if they were pretreated with 15-epi-lipoxin A_4_, suggesting that treatment with 15-epi-lipoxin A_4_ may be able to protect the fetus from the negative effects of the local inflammatory response induced by the surgery.

The mechanism by which 15-epi-lipoxin A_4_ reduces perinatal mortality in our model is currently unclear, although our data implicate prostanoid regulation via increased *Ptgs2* and decreased *15-Hpgd* expression in the uterus and placenta. This increased expression of *Ptgs2* could result in increased production of prostaglandins with anti-inflammatory effects, such as PGE_2_, PGD_2_ and 15d-PGJ_2_, as has been described in other studies (Gilroy *et al.*, 1999; Hodges *et al.*, 2004; Fukunaga *et al.*, 2005; Bonnans *et al.*, 2006; Zheng *et al.*, 2011; Font-Nieves *et al.*, 2012). These prostaglandins may act to resolve the inflammatory environment surrounding the fetus, thus leading to the reduced pup mortality rate observed in mice treated with 15-epi-lipoxin A_4_. Support for this hypothesis comes from a study that reported that administration of 15d-PGJ_2_ increased pup survival in a mouse model of LPS-induced PTL (Pirianov *et al.*, 2009).

Another potential mechanism by which 15-epi-lipoxin A_4_ could be acting to reduce pup mortality may be by promoting fetal lung maturation. PGE_2_ has been implicated in regulating fetal pulmonary surfactant production both *in vitro* (Acarregui *et al.*, 1990) and *in vivo* in a sheep model of intra-amniotic infection (Westover *et al.*, 2012); suggesting that the 15-epi-lipoxin A_4_-induced increase in utero-placental *Ptgs2* expression may promote fetal lung maturation via increased local PGE_2_ production. Additionally, a recent study reported that administration of a synthetic analogue of 15-epi-lipoxin A_4_ restored expression of surfactant protein C in lung tissue in a model of bleomycin-induced pulmonary fibrosis (Guilherme *et al.*, 2013); supporting the hypothesis that lipoxin administration can regulate lung surfactant production. Further work examining the inflammatory response at several time points is required to elucidate the relationship between *Ptgs2* and 15-epi-lipoxin A_4_ in our model, and to identify whether alterations in prostanoid production are involved in the reduced pup mortality observed in this study.

Interestingly, the administration of low-dose aspirin to women during pregnancy has been associated with reduced perinatal death and other adverse perinatal outcomes (Bujold *et al.*, 2010; Roberge *et al.*, 2013). As 15-epi-lipoxins are produced in the presence of aspirin, it is possible that 15-epi-lipoxin A_4_ is involved in mediating any beneficial effects of aspirin treatment. Other studies have shown that low-dose aspirin administration to healthy volunteers leads to significantly elevated plasma levels of 15-epi-lipoxin A_4_ (Chiang *et al.*, 2004), therefore, it would be interesting to assess whether similar mechanisms are acting during pregnancy.

Another important observation from our work which is worthy of further investigation is the finding that elevated levels of *Ptgs2* were also observed in uterus and placental tissue obtained from mice treated with 15-epi-lipoxin A_4_ alone, even though mice in this treatment group did not go into PTL. Previous studies have demonstrated a central role for elevated *Ptgs2* expression, and subsequent production of prostaglandins such as PGF_2α_ and PGE_2_ in the onset of parturition in mice (Sugimoto *et al.*, 1997; Gross *et al.*, 1998, 2000; Tsuboi *et al.*, 2003). However, mice in the 15-epi-lipoxin A_4_ group delivered at term, despite having elevated *Ptgs2* expression, again suggesting that treatment with 15-epi-lipoxin A_4_ may be triggering an alternative prostanoid pathway, as has been reported in other systems (Zheng *et al.*, 2011).

Interestingly, 15-epi-lipoxin A_4_ was unable to attenuate LPS-induced pro-inflammatory signalling in our model, which is in contrast to our previous work showing that lipoxin treatment *in vitro* attenuated *IL-6* and *IL-8* expression in human myometrial explant culture (Maldonado-Perez *et al.*, 2010). The reasons for these differences are unclear, but may be a result of differences in the type and dose of lipoxin used in the two studies, and also the time-point at which tissues were collected from our *in vivo* model. Perhaps if tissues had been collected at a different time-point, we may have observed alterations in inflammatory signalling. Whilst it is often difficult to extrapolate between animal models and the clinical scenario in humans, importantly, our *in vitro* data suggests that lipoxin treatment may have a more profound impact on inflammatory signalling in human tissues.

This study demonstrates for the first time that 15-epi-lipoxin A_4_ reduces pup mortality in a mouse model of LPS-induced PTL. Although the mechanisms by which 15-epi-lipoxin A_4_ may be acting to protect the prematurely delivered pups from mortality are not currently clear, we propose that 15-epi-lipoxin A_4_ may be stimulating the resolution of the LPS-induced inflammatory and/or promoting fetal maturation via increased *Ptgs2* expression and decreased *15-Hpgd* expression in the utero-placental tissues. Collectively, these data suggest that lipoxins warrant further investigation as potential novel therapeutic options in the treatment of PTL, which may be useful in protecting the fetus from the adverse effects of infection-induced preterm birth.

## Authors' roles

S.F.R., R.D.C. and J.W. performed the experiments. S.F.R. wrote the manuscript. S.F.R., R.D.C., J.W., A.G.R. and J.E.N. contributed to the design of the study, analysis and interpretation of the data, drafting of the article and final approval of the version to be published.

## Funding

This work was supported by grants from Tommy's the baby charity and PiggyBank Kids. S.F.R. is supported by Medical Research Council (grant number MR/L002657/1). Funding to pay the Open Access publication charges for this article was provided by the Medical Research Council.

## Conflict of interest

No authors declare any financial or other relationships that might lead to a conflict of interest.
